# VUV spectroscopy of an electron irradiated benzene : carbon dioxide interstellar ice analogue

**DOI:** 10.1039/c9ra00462a

**Published:** 2019-02-13

**Authors:** Rachel L. James, Nykola C. Jones, Søren V. Hoffmann, Anita Dawes

**Affiliations:** School of Physical Sciences, The Open University Walton Hall, Milton Keynes UK Rachel.James1@open.ac.uk +44(0) 1908 654192 +44(0) 1908 332012; ISA, Centre for Storage Ring Facilities, Department of Physics and Astronomy, Aarhus University Ny Munkegade 120, DK-8000 Aarhus C Denmark

## Abstract

We present the first vacuum ultraviolet spectroscopic study of an interstellar ice analogue of a 1 : 100 benzene (C_6_H_6_) : carbon dioxide (CO_2_) mixture which has been energetically processed with 1 keV electrons. We have exploited the fact that benzene has a relatively high photoabsorption cross section in the vacuum ultraviolet region to study this dilute mixture of benzene. Before irradiation with 1 keV electrons, we observed that the benzene electronic transition bands in the C_6_H_6_ : CO_2_ mixture exhibits a blueshift in band position towards energies observed in the gas-phase compared with that of pure, amorphous benzene and we have attributed this to a matrix isolation effect. After irradiation, a lowering in intensity of both the carbon dioxide and benzene electronic transition bands was observed, as well as the formation of the small irradiation product, carbon monoxide. A residue was obtained at 200 K which showed characteristic features of the benzene electronic transition of ^1^E_1u_ ← ^1^A_1g_, but with additional structure suggesting the formation of a benzene derivative.

## Abstract 2

We present the first vacuum ultraviolet spectroscopic study of an interstellar ice analogue of a benzene (C_6_H_6_) : carbon dioxide (CO_2_) (1 : 100) mixture which has been energetically processed with 1 keV electrons.

## Graphical abstract

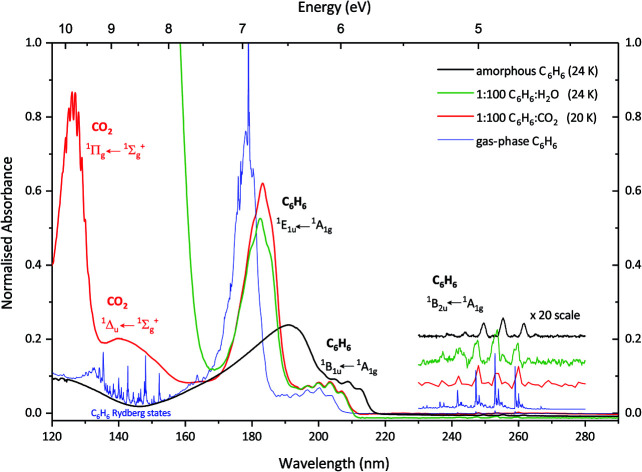

## 1 Introduction

Polycyclic aromatic hydrocarbons (PAHs) have attracted considerable attention in the astrochemistry community due to their ubiquitous presence in the interstellar medium (ISM). It is thought that up to 10% of the interstellar carbon budget is in the form of PAHs^1^ and absorption features around young stellar objects contain characteristic aromatic C–C and C–H stretches suggesting the presence of condensed phase PAHs in icy mantles.^2^ However, despite the interest surrounding PAHs there still has not been a single PAH identified in the ISM. The structure of most PAHs makes them difficult to observe using rotational spectroscopy and observations with infrared spectroscopy generally lack the resolution to distinguish between different PAHs.

Nevertheless, benzene, a prototypical aromatic molecule and building block of PAHs, has been detected in the gas-phase in the protoplanetary nebula CRL 618 ^3^ and the Large Magellanic Cloud object SMP LMC 11.^4^ Recently a derivative of benzene, benzonitrile, was detected in the gas-phase in the molecular cloud TMC-1.^5^ Formation routes for PAHs in the diffuse ISM include the top-down processes of SiC dust grain destruction from the intense UV radiation field and sputtering from shockwaves.^6,7^ Alternative bottom-up processes include the formation of PAHs from benzene derivatives or radicals in the gas-^8^ or solid-phase which are more likely in low temperature, molecular cloud environments. Within our own Solar System, PAHs and benzene derivatives have been detected on some Saturnian moons,^9^ interplanetary dust grains^10^ and meteorites. In fact, some of the PAHs and benzene derivatives detected in meteoritic materials show deuterium enrichment indicating that the formation origin of these molecules may be from molecular cloud environments.^11^

Although PAHs, benzene and its derivatives have not been directly identified in the solid-phase, which is most likely due to their weak infrared absorption features overlapping with intense ice absorption bands, they are believed to be incorporated into icy mantles around interstellar dust grains or operate as nucleation sites for the condensation of other species.^12^ Within molecular clouds, the icy mantles of interstellar dust grains may be subjected to energetic processing (protons, secondary UV photons or secondary electrons) which could induce molecular synthesis within the ice. Several experimental studies have looked at the formation of PAHs or benzene derivatives within interstellar ice analogues.^13–15^ In particular, aromatic molecules with the carboxylic acid moiety (–COOH) have been studied because of their role as precursors to amino acids with non-aromatic carboxylic acids detected in the ISM in the form of formic acid (HCOOH)^16^ and acetic acid (CH_3_COOH).^17^ Carboxylic acids with varying degrees of complexity have also been detected in the Tagish Lake,^18–20^ Murchison^21,22^ and Orgueil^22,23^ carbonaceous meteorites.

In this paper we investigate the energetic processing of a dilute mixture of benzene in CO_2_, as an interstellar ice analogue, with 1 keV electrons using vacuum ultraviolet (VUV) spectroscopy. While mid-IR spectroscopy is the more commonly used *in situ* laboratory technique for investigating the structure and composition of interstellar ices, and any changes upon energetic processing, this is the first time that VUV spectroscopy has been used to study the benzene : CO_2_ system. With VUV spectroscopy we can take advantage of the fact that benzene has appreciatively high photoabsorption cross sections due to strong electronic π → π* transitions compared to that of other interstellar ice constituents such as H_2_O and CO_2_. For example, cross sections of benzene at wavelengths <200 nm reach 50 Mb ^24^ which is almost 40 times more intense compared to that of the CO_2_ peak cross section of around 1.3 Mb.^25^ In contrast, the mid-IR band strength (*A*-value) of the strongest vibrational transition of solid benzene, the C–C stretch (*v*_19_), is 1.4 × 10^−18^ cm per molecule^26^ which is approximately 90 times less intense compared to the C–O stretch (*v*_3_) of CO_2_ with a band strength of 1.3 × 10^−16^ cm per molecule.^27^ This makes VUV spectroscopy a potentially more favourable region of the electromagnetic spectrum to study very dilute mixtures of benzene in ice matrices (*e.g.* benzene and water). In this paper we present the results of the first experimental study using VUV spectroscopy to investigate an electron irradiated, 1 : 100 C_6_H_6_ : CO_2_ mixture as an interstellar ice analogue.

## 2 Experimental

Experiments were carried out using The Open University's Portable Astrochemistry Chamber (PAC) attached to the AU-UV beamline at the ASTRID2 Synchrotron Facility in Aarhus, Denmark.^28^ This set-up has been used before for the investigation of condensed phase molecules and is described in detail elsewhere by Dawes *et al.*^24^ and only a brief description is given here.

The experiments were carried out at a base pressure of low 10^−8^ mbar. Benzene (anhydrous; Sigma-Aldrich, 99.8% purity) was degassed *via* three freeze–pump–thaw cycles prior to use and pre-mixed with CO_2_ (Sigma-Aldrich, 99.995% purity) in the gas line. The mixture was then vapour deposited onto a cold (20 K) MgF_2_ (Crystran) substrate held in a copper holder attached to a closed cycle helium cryostat (Sumitomo). VUV spectra were acquired in the range 120–340 nm with 0.05 to 1 nm wavelength step size depending on the width of the spectral features to be resolved. Absorbance spectra were calculated from the recorded incident and transmitted intensities as a function of wavelength (nm).

Film thickness was determined from *in situ* laser interferometry measurements from the use of a HeNe laser beam reflected off the substrate during deposition. Deposition rates were between 0.12 and 0.55 nm s^−1^ depending on the thickness of the ice being grown. The final thickness of the films were 349 nm for the unirradiated mixture and a 1.28 μm for the irradiated mixture. For the unirradiated mixture a thinner ice was prepared to avoid saturation of the electronic transition bands, to allow for accurate band assignments and observe any changes in the band profiles upon annealing. A thicker ice was prepared for irradiation to ensure a sufficient quantity of products were formed for observation/monitoring and to ensure that the ice thickness was greater than the penetration depth of electrons so that there was no substrate effect. This thicker ice was grown in stages to confirm that no thickness-dependent effects were present until saturation of the peaks was observed.

The mixture was irradiated with 1 keV electrons (Kimball Physics FRA-2X1-2/EGPS-1011A) with a current of 10 μA and the bombarded area was about 3.5 cm^−2^. The electron flux at the sample was approximately 1.77 × 10^13^ electrons per cm^2^ per s. After 180 min of irradiation this corresponded to a fluence of 1.91 × 10^17^ electrons per cm^2^. A penetration depth of 40 nm for 1 keV electrons was estimated using Monte Carlo simulations from the CASINO code.^29^

Samples were annealed gradually at a rate of 1 K min^−1^ by heating the sample using a Kapton heater and regulated with a temperature controller (Oxford Instruments). The samples were held at the required temperature for 2 min and then returned to base temperature (20 K) prior to measuring the spectra.

## 3 Results and discussion

From previous studies^24,30^ we know that when solid-phase benzene is diluted with amorphous solid water a blueshift in the electronic bands compared to that of pure benzene is observed. We therefore used an unirradiated control sample to characterise the 1 : 100 C_6_H_6_ : CO_2_ mixture and any effects annealing might produce, the results of which are discussed in Section 3.1. In Section 3.2 we discuss the results of irradiating the 1 : 100 C_6_H_6_ : CO_2_ mixture with 1 keV electrons for 180 min and in Section 3.2.1 we discuss the results on the residue left at 200 K.

### 3.1 Characterisation of an unirradiated 1 : 100 C_6_H_6_ : CO_2_ mixture

#### 3.1.1 The electronic structure of a 1 : 100 C_6_H_6_ : CO_2_ mixture at 20 K

**Fig. 1:**
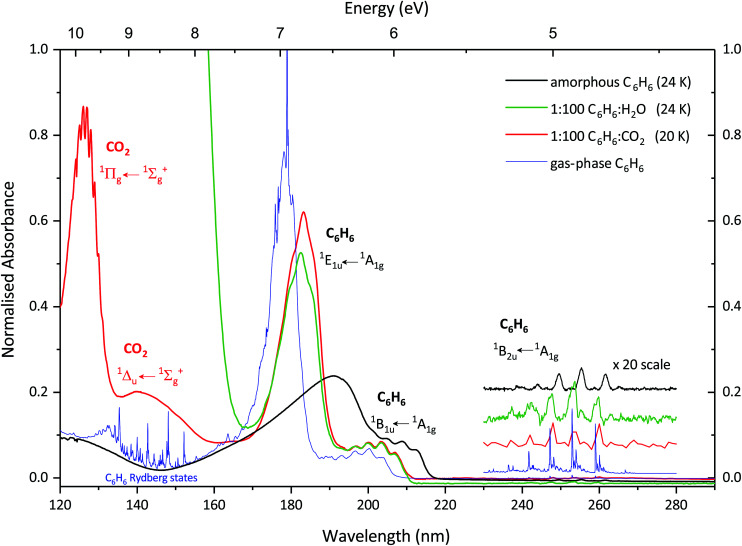
VUV photoabsorption spectra of 1 : 100 C_6_H_6_ : CO_2_ mixture deposited at 20 K (red) compared with pure, amorphous benzene deposited at 24 K (black), 1 : 100 C_6_H_6_ : H_2_O mixture deposited at 24 K (green) and gas-phase benzene (blue) from ref. 24 and 30. The band assignments and positions are summarised in Table 1. The ^1^B_2u_ ← ^1^A_1g_ vibronic bands between 230–270 nm have been scaled *x* 20 and offset for clarity. The spectra are normalised to the integrated area of the overlapping ^1^E_1u_ ← ^1^A_1g_ and ^1^B_1u_ ← ^1^A_1g_ transitions of the 1 : 100 C_6_H_6_ : CO_2_ mixture deposited at 20 K.

Fig. 1 shows the VUV photoabsorption spectrum of a 1 : 100 C_6_H_6_ : CO_2_ mixture deposited at 20 K compared with spectra of a 1 : 100 C_6_H_6_ : H_2_O mixture deposited at 24 K,^30^ pure benzene deposited at 24 K and gas-phase benzene.^24^ Two broad bands centred around 127 and 142 nm are due to CO_2_ and correspond to the 
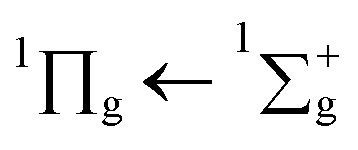
 and 
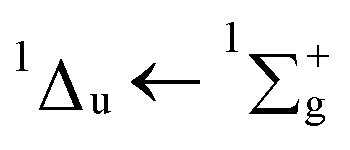
 transitions respectively. The higher energy 
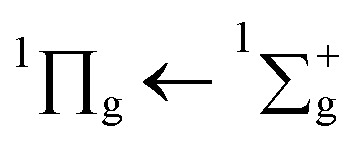
 transition exhibits vibronic structure the positions of which are in good agreement (to within 10 meV) with a study conducted by Mason *et al.*^25^ where pure CO_2_ was deposited at 20 K.

Three benzene bands in the 1 : 100 C_6_H_6_ : CO_2_ mixture are observed in Fig. 1 and correspond to the ^1^E_1u_ ← ^1^A_1g_, ^1^B_1u_ ← ^1^A_1g_ and ^1^B_2u_ ← ^1^A_1g_ transitions. The ^1^E_1u_ ← ^1^A_1g_ transition is the most intense benzene band and it is blueshifted by 0.30 eV from 191.8 nm (6.47 eV) in pure benzene to 183.2 nm (6.77 eV) in the 1 : 100 C_6_H_6_ : CO_2_ mixture. The ^1^B_1u_ ← ^1^A_1g_ transition appears as a structured shoulder on the long wavelength side of the ^1^E_1u_ ← ^1^A_1g_ transition (assignments are given in Table 1) and is again blueshifted compared to that of pure benzene. Interestingly the benzene band assignments for the 1 : 100 C_6_H_6_ : CO_2_ mixture are in good agreement with a 1 : 100 C_6_H_6_ : H_2_O mixture. In the previous study by Dawes *et al.*^30^ several ratios (1 : 1, 1 : 10 and 1 : 100) of C_6_H_6_ : H_2_O were studied and as the benzene was diluted, successive blueshifts from pure benzene were observed and attributed to a matrix isolation effect. Also, the 1 : 100 C_6_H_6_ : H_2_O mixture showed structure within in the ^1^E_1u_ ← ^1^A_1g_ transition band which resembled the band profile of gas-phase benzene and was not observed in the less diluted benzene mixtures. This gas-phase like band profile is also observed in the 1 : 100 C_6_H_6_ : CO_2_ mixture of the ^1^E_1u_ ← ^1^A_1g_ transition band and further supports the theory of matrix isolation as a cause of the shifts observed in the electronic band rather than a solvent effect. While the vibronic peaks of the ^1^B_2u_ ← ^1^A_1g_ electronic transition are not completely resolved, the data is sufficient to suggest a blue shift in position towards energies observed in the gas-phase in a similar fashion to that of the 1 : 100 C_6_H_6_ : H_2_O mixture.

**Table 1:** Band assignments and positions of the main electronic and vibronic bands for benzene shown in Fig. 1 for a 1 : 100 C_6_H_6_ : CO_2_ mixture deposited at 20 K, annealed to 80 K and then 90 K compared with pure benzene at 24 K and gas-phase benzene from ref. 24 and 30. The gas to solid energy shifts (in eV) are shown under headings ‘g–s’

Band assignment	C_6_H_6_ (solid, 20 K)^24^			1 : 100 C_6_H_6_ : CO_2_			1 : 100 C_6_H_6_ : H_2_O^30^			C_6_H_6_ (gas)^24^	
	nm	eV	g–s/eV	nm	eV	g–s/eV	nm	eV	g–s/eV	nm	eV
^**1**^ **E**_**1u**_ **←** ^**1**^ **A**_**1g**_											
	191.8	6.47	0.50	183.2	6.77	0.20	182.7	6.79	0.18	178.0	6.97

^**1**^ **B**_**1u**_ **←** ^**1**^ **A**_**1g**_											
6^1^_0_	212.7	5.83	0.24	207.0	5.99	0.08	207.0	5.99	0.08	204.2	6.07
6^1^_0_1^1^_0_	208.8	5.94	0.25	203.4	6.10	0.09	203.6	6.09	0.10	200.3	6.19
6^1^_0_1^2^_0_	204.9	6.05	0.26	200.2	6.19	0.12	200.1	6.20	0.11	196.6	6.31
6^1^_0_1^3^_0_	201.2	6.16	0.26	196.8	6.30	0.12	196.5	6.31	0.11	193.0	6.42
6^1^_0_1^4^_0_				193.4	6.41	0.12	193.3	6.41	0.12	189.8	6.53

#### 3.1.2 The electronic structure of a 1 : 100 C_6_H_6_ : CO_2_ mixture annealed to 80, 90 and 140 K

**Fig. 2:**
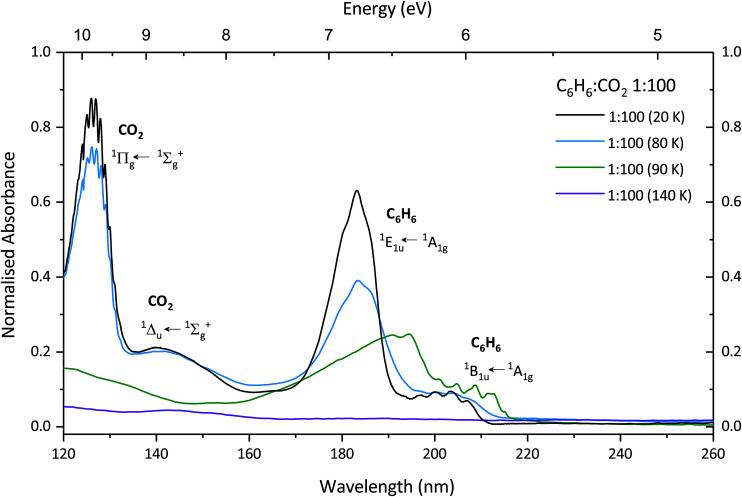
VUV photoabsorption spectra of a 1 : 100 C_6_H_6_ : CO_2_ mixture deposited at 20 K (black) and annealed to 80 K (blue), 90 K (green) and 140 K (purple). The spectra are normalised to the integrated area of the overlapping ^1^E_1u_ ← ^1^A_1g_ and ^1^B_1u_ ← ^1^A_1g_ transitions of the 1 : 100 C_6_H_6_ : CO_2_ mixture deposited at 20 K.

Fig. 2 shows the VUV photoabsorption spectra of the 1 : 100 C_6_H_6_ : CO_2_ mixture as it is annealed to 80, 90 and 140 K corresponding to the temperature at which pure CO_2_ is crystalline (>80 K), pure benzene begins to crystallise and pure CO_2_ desorbs (75–90 K), and, pure benzene desorbs (140 K). At 80 K the CO_2_
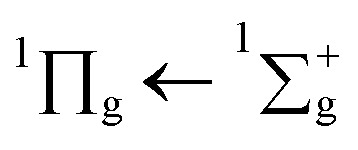
 and 
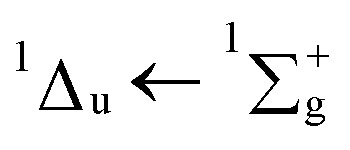
 transition bands are still present in the same position as that of spectra taken at 20 K, albeit with lower intensity, and by 90 K they have disappeared due to desorption of CO_2_. At 80 K the ^1^E_1u_ ← ^1^A_1g_ transition of benzene has lowered in intensity and the band profile is showing signs of the splitting characteristic of that of crystallisation of benzene. The ^1^B_1u_ ← ^1^A_1g_ vibronic structure appears less well defined than at 20 K and may be due to desorption of CO_2_ which is also occurring at 80 K. When the CO_2_ is fully desorbed at 90 K the ^1^E_1u_ ← ^1^A_1g_ has shifted from 183.4 nm to 192.8 nm with a well defined splitting in the band (190.6 nm and 194.6 nm) observed. Additionally the ^1^B_1u_ ← ^1^A_1g_ vibronic features have sharpened compared to that at 80 K and shifted to pure, crystalline benzene positions.^24^ At 140 K all the benzene has been removed from the substrate.

### 3.2 Electron irradiation and subsequent annealing of a 1 : 100 C_6_H_6_ : CO_2_ mixture

**Fig. 3:**
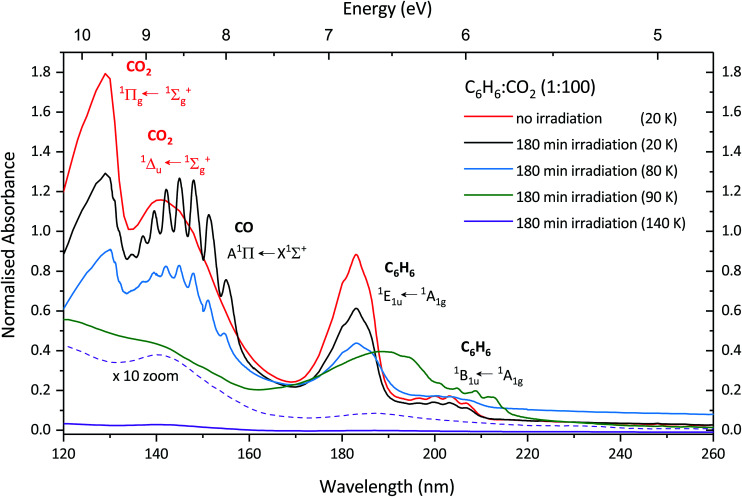
VUV photoabsorption spectra of a 1 : 100 C_6_H_6_ : CO_2_ mixture deposited at 20 K (red), irradiated for 180 min with 1 keV electrons (black) and annealed to 80 K (blue), 90 K (green) and 140 K (purple). The purple dashed line shows the 140 K trace scaled × 10. The spectra are normalised to the integrated area of the overlapping ^1^E_1u_ ← ^1^A_1g_ and ^1^B_1u_ ← ^1^A_1g_ transitions of the 1 : 100 C_6_H_6_ : CO_2_ mixture deposited at 20 K.

After irradiating with 1 keV electrons there is a noticeable difference in the spectrum compared to that of the unirradiated spectrum as shown in Fig. 3. The irradiation product carbon monoxide (CO) is observed *via* the vibronic structure of the A^1^Π ← X^1^∑^+^ transition, the assignments are shown in Table 2 and compared with pure CO and CO formed *via* VUV irradiation of CO_2_. Davydov splitting observed in the lower energy bands (0,0–3,0) of pure CO are not observed in CO formed from irradiation of electrons, consistent with that of the CO formed from VUV photons in a study by Cruz-Diaz *et al.*^31^ Additionally the vibronic bands have blueshifted compared to that of pure CO, which is also consistent with that of Cruz-Diaz *et al.*^31^ The 
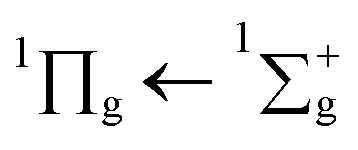
 transition of CO_2_ is also present after irradiation but at lower intensity, which might be due to a combination of dissociation from electron irradiation and sputtering. The 
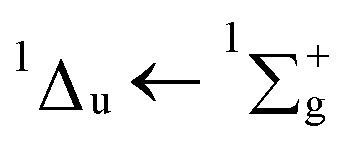
 transition of CO_2_ is not observable due to the overlapping A^1^Π ← X^1^∑^+^ CO band which has a much higher cross section. The ^1^E_1u_ ← ^1^A_1g_ and ^1^B_1u_ ← ^1^A_1g_ transitions of benzene are also present in the same position as the unirradiated benzene, albeit with lower intensity due to formation of products or sputtering.

**Table 2:** Band assignments and positions of the CO A^1^Π ← X^1^∑^+^ transition formed from a 1 : 100 C_6_H_6_ : CO_2_ mixture irradiated by 1 keV electrons for 180 min and compared with pure CO taken from Mason *et al.*^25^and CO formed from VUV photons in a CO_2_ matrix.^31^ Band splitting is indicated by numbers in parenthesis

Band assignment (*v*′, *v*′′)	Pure CO^25^	This work		CO in CO_2_ matrix^31^	
	eV	nm	eV	nm	eV
7, 0	9.16	134.75	9.20	134.8	9.20
6, 0	9.01	137.10	9.04	137.0	9.05
5, 0	8.88	139.60	8.88	139.4	8.89
4, 0	8.68	142.10	8.73	142.1	8.73
3, 0	8.48 (8.51)	144.95	8.55	144.8	8.56
2, 0	8.29 (8.33)	148.00	8.38	147.9	8.38
1, 0	8.09 (8.14)	151.35	8.19	151.1	8.21
0, 0	7.90 (7.96)	155.00	8.00	154.4	8.03

Upon annealing to 80 K there is a further decrease in intensity of the 
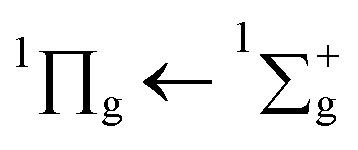
 transition of CO_2_ as it is close to the desorption temperature of CO_2_. At 80 K it is expected that the CO would have desorbed, as pure CO desorbs between 28–36 K depending on the original deposition temperature (a lower deposition temperature will allow H_2_ to vapour deposit on the surface which can stimulate desorption at lower temperatures).^32^ However, the A^1^Π ← X^1^∑^+^ transition is still clearly observable although the intensity is lowered somewhat, with the (7,0) band no longer visible and the (6,0) band less well defined. Like the unirradiated sample discussed in Section 3.1.2 the benzene transition bands are present with a lower intensity for both electronic transitions and the vibronic structure of the ^1^B_1u_ ← ^1^A_1g_ transition are less well defined compared to that of spectrum taken at 20 K. At 90 K CO_2_ and CO have desorbed and interestingly the benzene band has increased in intensity. The ^1^E_1u_ ← ^1^A_1g_ transition shows signs of splitting similar to that observed in the unirradiated sample indicating crystallisation of benzene, however, this is less well defined compared to that of the unirradiated sample and may be due to the bands in the sample being saturated as the sample is thicker than the unirradiated sample. At 140 K the spectrum looks relatively flat; however, when zoomed in the ^1^E_1u_ ← ^1^A_1g_ transition is present, indicating that benzene is still desorbing at this temperature.

#### 3.2.1 Residue of a 1 : 100 C_6_H_6_ : CO_2_ electron irradiated mixture

**Fig. 4:**
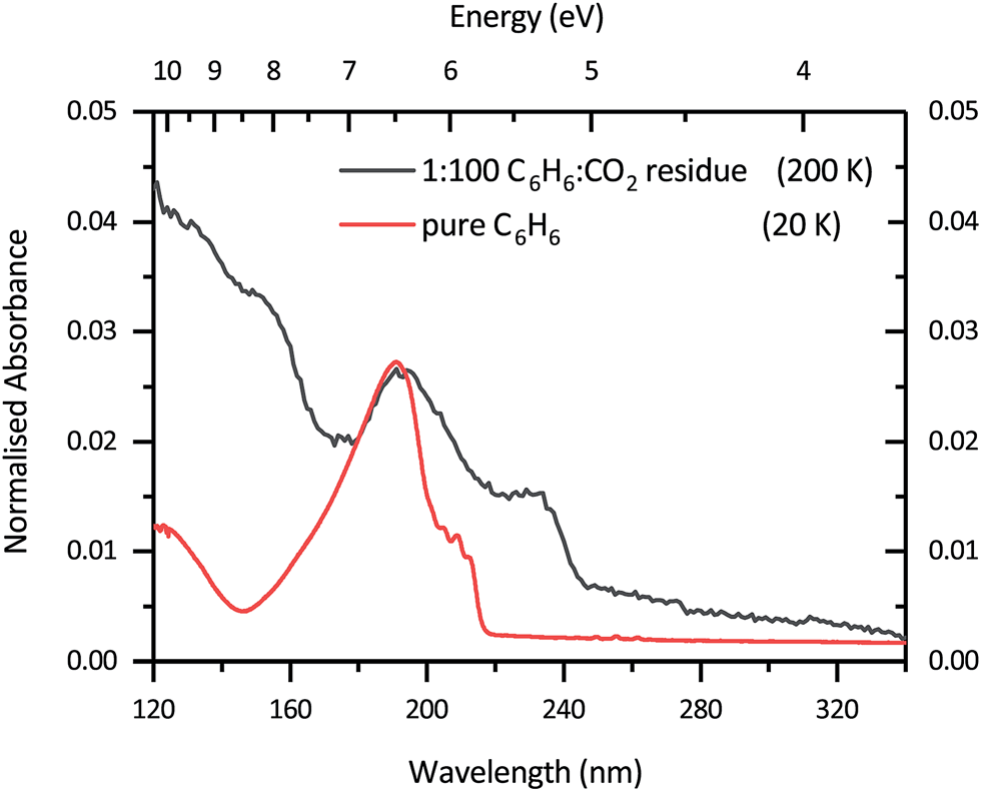
VUV photoabsorption spectra of a 1 : 100 C_6_H_6_ : CO_2_ mixture which was irradiated with 1 keV electrons for 180 min and annealed to 200 K (black) to form a residue compared with pure benzene (red) at 20 K. The pure benzene spectrum at 20 K has been normalised to the peak height at *λ* = 190 nm of the 1 : 100 C_6_H_6_ : CO_2_ residue.

By 200 K all of the benzene has desorbed and a residue is left as shown in Fig. 4 and while it displays spectral characteristics similar to that of benzene it has additional structure around 230 nm not typical of pure benzene. From our work, yet to be published,^33^ on toluene, which is a derivative of benzene, we know that the electronic transitions of toluene redshift compared to that of benzene. Additionally, a study by Hashimoto and Akimoto^34^ showed that when UV spectra of several derivatives of benzene (toluene, p-xylene, mesitylene and durene) in oxygen matrices were taken the absorption maxima of the absorption bands shift from 238 nm for benzene to 266 nm for durene (the most substituted ring). This may suggest that the additional feature at 230 nm is due to the addition of a functional group on the benzene ring, in this case a carboxyl group. Furthermore, the mid-IR results obtained in the study by McMurtry *et al.*^15^ at 180 K show evidence of benzoic acid, isophthalic acid and terephthalic acid present, which also leads us to believe that our residue at 200 K contains benzene derivatives. However, further studies on benzene derivatives in the VUV are still required for more definitive identification of products as such data, to our knowledge, is not available in the literature.

## 4 Conclusion

We have presented the results of the first vacuum ultraviolet spectroscopic study of the interstellar ice analogue of a 1 : 100 C_6_H_6_ : CO_2_ mixture. Benzene is a prototypical, aromatic molecule believed to be present in the icy mantles of interstellar dust where simple molecules such as H_2_O, CO, CO_2_, CH_4_ and NH_3_ are known to reside. These icy mantles will be subjected to energetic processing which can alter the composition of the icy mantles and possibly form benzene derivatives which may later be released into the gas-phase through thermal or photo-induced desorption. Benzene has a relatively high photoabsorption cross section in the vacuum ultraviolet part of the electromagnetic spectrum making it an attractive spectroscopic technique to use for the study of interstellar ice analogues containing dilute mixtures of benzene. Characterisation of the interstellar ice analogue before irradiation suggests that when benzene is diluted into CO_2_ a matrix-isolation effect takes place. This is evidenced through the shift in the ^1^E_1u_ ← ^1^A_1g_ and ^1^B_1u_ ← ^1^A_1g_ electronic transition towards gas-phase values of benzene compared to that of pure, solid benzene and the change in band profile to resemble a more gas-like character. Benzene derivatives are predicted to form after irradiation of the interstellar ice analogue^15^ and the residue at 200 K observed in this study shows that a benzene-like structure has formed. However, definitive assignment begs the need for further VUV spectroscopic work of condensed molecular films of potential irradiation products formed as such data has not yet been published.

## Conflicts of interest

There are no conflicts to declare.

## Supplementary Material

## References

[cit1] Chiar J., Tielens A. G. G. M., Adamson A., Ricca A. (2013). Astrophys. J..

[cit2] Keane J. V., Tielens A. G. G. M., Boogert A. C. A., Schutte W. A., Whittet D. C. B. (2001). Astron. Astrophys..

[cit3] Cernicharo J., Heras A. M., Tielens A. G. G. M., Pardo J. R., Herpin F., Guélin M., Waters L. B. F. M. (2001). Astrophys. J..

[cit4] Malek S. E., Cami J., Bernard-Salas J. (2011). Astrophys. J..

[cit5] McGuire B. A., Burkhardt A. M., Kalenskii S., Shingledecker C. N., Remijan A. J., Herbst E., McCarthy M. C. (2018). Science.

[cit6] Merino P., Švec M., Martinez J., Jelinek P., Lacovig P., Dalmiglio M., Lizzit S., Soukiassian P., Cernicharo J., Martin-Gago J. (2014). Nat. Commun..

[cit7] Zhao T. Q., Li Q., Liu B. S., Gover R. K. E., Sarre P. J., Cheung A. S.-C. (2016). Phys. Chem. Chem. Phys..

[cit8] Parker D. S. N., Zhang F., Kim Y. S., Kaiser R. I., Lander A., Kislov V. V., Mebel A. M., Tielens A. G. G. M. (2012). Proc. Natl. Acad. Sci. U. S. A..

[cit9] Cruikshank D. P., Ore C. M. D., Clark R. N., Pendleton Y. J. (2014). Icarus.

[cit10] Clemett S. J., Maechling C. R., Zare R. N., Swan P. D., Walker R. M. (1993). Science.

[cit11] Sandford S. A. (2002). Planet. Space Sci..

[cit12] Geers V. C., van Dishoeck E. F., Pontoppidan K. M., Lahuis F., Crapsi A., Dullemond C. P., Blake G. A. (2009). Astron. Astrophys..

[cit13] Smith K. E., Callahan M. P., Gerakines P. A., Dworkin J. P., House C. H. (2014). Geochim. Cosmochim. Acta.

[cit14] Materese C. K., Nuevo M., Sandford S. A. (2015). Astrophys. J..

[cit15] McMurtry B. M., Saito S. E. J., Turner A. M., Chakravarty H. K., Kaiser R. I. (2016). Astrophys. J..

[cit16] Zuckerman B., Ball J., Gottlieb C. A. (1971). Astrophs. J..

[cit17] Mehringer D. M., Snyder L. E., Miao Y., Lovas F. J. (1997). Astrophs. J..

[cit18] Pizzarello S., Huang Y., Becker L., Poreda R. J., Nieman R. A., Williams G. C. M. (2001). Science.

[cit19] Pizzarello S., Huang Y. (2002). Meteorit. Planet. Sci..

[cit20] Hilts R. W., Herd C. D. K., Simkus D. N., Slater G. F. (2014). Meteorit. Planet. Sci..

[cit21] Pizzarello S., Huang Y. (2005). Geochim. Cosmochim. Acta.

[cit22] Martins Z., Watson J. S., Sephton M. A., Botta O., Ehrenfreund P., Gilmour I. (2006). Meteorit. Planet. Sci..

[cit23] Remusat L., Derenne S., Robert F. (2005). Geochim. Cosmochim. Acta.

[cit24] Dawes A., Pascual N., Hoffmann S. V., Jones N. C., Mason N. J. (2017). Phys. Chem. Chem. Phys..

[cit25] Mason N. J., Dawes A., Holtom P. D., Mukerji R. J., Davis M. P., Sivaraman B., Kaiser R. I., Hoffmann S. V., Shaw D. A. (2006). Faraday Discuss..

[cit26] Zhou L., Zheng W., Kaiser R. I., Landera A., Mebel A. M., Liang M.-C., Yung Y. L. (2010). Astrophs. J..

[cit27] Bouilloud M., Fray N., Bénilan Y., Cottin H., Gazeau M.-C., Jolly A. (2015). Mon. Not. R. Aston. Soc..

[cit28] Palmer M. H., Ridley T., Hoffmann S. V., Jones N. C., Coreno M., de Simone M., Grazioli C., Biczysko M., Baiardi A., Limão-Vieira P. (2015). J. Chem. Phys..

[cit29] Drouin D., Couture A. R., Joly D., Tastet X., Aimez V., Gauvin R. (2007). Scanning.

[cit30] Dawes A., Pascual N., Mason N. J., Gärtner S., Hoffmann S. V., Jones N. C. (2018). Phys. Chem. Chem. Phys..

[cit31] Cruz-Diaz G., Caro G. M. M., Chen T.-J., Yih T.-S. (2014). Astron. Astrophys..

[cit32] Caro G. M. M., Jiménez-Escobar A., Martín-Gago J. Á., Rogero C., Atienza C., Puertas S., Sobrado J. M., Torres-Redondo J. (2010). Astron. Astrophys..

[cit33] James R. L. , Dezaley J., Jones N. C., Hoffmann S. V. and Dawes A., unpublished work

[cit34] Hashimoto S., Akimoto H. (1989). J. Phys. Chem..

